# Antioxidant Properties and Antinutritional Components of Flowers from Five Pumpkin Species

**DOI:** 10.3390/antiox14111353

**Published:** 2025-11-12

**Authors:** Małgorzata Stryjecka, Tomasz Cebulak, Barbara Krochmal-Marczak, Anna Kiełtyka-Dadasiewicz

**Affiliations:** 1 Institute of Human Nutrition and Agriculture, The University College of Applied Sciences in Chełm, 22-100 Chełm, Poland; mstryjecka@panschelm.edu.pl; 2 Department of Food Technology and Human Nutrition, Institute of Food Technology and Nutrition, College of Natural Sciences, University of Rzeszów, 35-601 Rzeszów, Poland; tcebulak@ur.edu.pl; 3 Department of Plant Production and Food Safety, The University College of Applied Sciences in Krosno, 38-400 Krosno, Poland; barbara.marczak@pans.krosno.pl; 4 Department of Plant Production Technology and Commodity Sciences, University of Life Sciences, 20-950 Lublin, Poland

**Keywords:** pumpkin flowers, antioxidant activity, total phenolics, carotenoids, flavonoids, β-carotene, *Cucurbita*, antinutritional compounds

## Abstract

The contents of total polyphenols, flavonoids, phenolic acids, anthocyanins, and carotenoids were determined using spectrophotometric and chromatographic methods, alongside antioxidant activity: 2,2-diphenyl-1-picrylhydrazyl (DPPH), Ferric Reducing Antioxidant Power (FRAP), Cupric Reducing Antioxidant Capacity (CUPRAC), and hydroxyl radical scavenging assays). Additionally, the levels of antinutritional compounds (tannins, phytates, oxalates, alkaloids, and saponins) were assessed in the flowers of five pumpkin species: giant pumpkin, summer squash, butternut squash, fig-leaf gourd, and cushaw squash (*Cucurbita maxima*, *C. pepo*, *C. moschata*, *C. ficifolia*, and *C. argyrosperma*). The results revealed significant interspecific variation in both bioactive and antinutritional compounds. Giant pumpkin flowers exhibited the highest content of polyphenols and phenolic acids, fig-leaf gourd flowers were the richest in carotenoids, whereas butternut squash flowers had the highest anthocyanin levels. The strongest antioxidant activity was observed in giant pumpkin flowers, which can be attributed to their high phenolic and flavonoid content. Despite the presence of moderate amounts of antinutritional compounds, pumpkin flowers can be considered a valuable edible raw material with nutraceutical potential.

## 1. Introduction

Pumpkin (*Cucurbita* spp.), belonging to the *Cucurbitaceae* family, is an annual plant cultivated worldwide, including in South America (Chile, Argentina), Europe, Asia (China, India), and the western regions of North America [1,2,3,4,5,6]. Pumpkin fruits are a rich source of carotenoids, pectins, minerals, vitamins, and numerous other bioactive compounds that contribute to the prevention of several civilization-related diseases, such as hypertension, type 2 diabetes, cancer [7,8,9,10,11], coronary heart disease [12], inflammatory bowel disease, dyspepsia and gastric disorders [4,13,14].

Traditionally, the most widely utilized parts of pumpkin have been its seeds and fruits. In recent years, however, the culinary and nutritional value of pumpkin flowers has been increasingly recognized. These flowers exhibit anti-inflammatory and antioxidant properties, are low in calories, and aid digestion [1,2,15]. Nevertheless, most previous studies on pumpkins have primarily focused on the health-promoting value of fruits [1,14,16], while the nutritional and functional properties of pumpkin flowers remain underexplored despite their high dietary potential.

According to Bieżanowska-Kopeć et al. [17], pumpkin flowers are a source of C, A, D, E, K, and B-complex vitamins, as well as minerals such as calcium, iron, potassium, and phosphorus. Consumption of these generative plant organs strengthens the cardiovascular and immune systems and positively influences vision and skin health. Traditionally, pumpkin flowers have been used in folk medicine as a remedy for male infertility, minor injuries, and mild fever, due to their richness in calcium, potassium, and sodium [18]. The presence of carotenoids and gallic acid confers anti-inflammatory and anticancer properties, respectively. Additionally, due to their high antioxidant activity, pumpkin flowers may enhance immune function and overall biological activity by reducing oxidative stress [18,19].

With the increasing consumer interest in edible flowers as innovative food products that can be consumed fresh, dried, candied, crystallized, or pickled, this study was undertaken to provide comprehensive insights into the antioxidant activity of edible flowers from five *Cucurbita* species cultivated under the same meteorological conditions.

## 2. Materials and Methods

### 2.1. Materials

The plant material used in the study consisted of flowers from five pumpkin species: giant pumpkin (*Cucurbita maxima* ‘Bambino’), summer squash (*C. pepo* ‘Kamo Kamo’), butternut squash (*C. moschata* ‘Butternut’), fig-leaf gourd (*C. ficifolia* ‘Chilacayote Squash’), and cushaw squash (*C. argyrosperma* ‘Chinese Alphabet’). The material originated from controlled experimental cultivation conducted by the authors, not from wild populations. The flowers were harvested from pumpkin plants cultivated in 2018 in Żyznów (49°49′ N, 21°50′ E; Poland) on slightly acidic brown soil (pH/KCl 5.66%). From each species, 40 flowers were randomly collected in July during the morning hours. The collected flowers were placed in perforated paper bags and transported to the laboratory within one hour. Flowers were carefully cleaned, ground using a laboratory mill, and subsequently lyophilized (Lyovapor L-250 Pro, BUCHI, Flawil, Switzerland). Pumpkins species confirmation was carried out by dr hab. inż. Anna Kiełtyka-Dadasiewicz with dr hab. inż. Barbara Krochmal-Marczak, based on morphological characteristics and cultivar names consistent with the *Cucurbitae* taxonomic classification.

### 2.2. Chemicals

All chemicals and solvents used in this study were of analytical or HPLC (high-performance liquid chromatography) grade witch purity levels ≥ 95–99%. The reagents were purchased from Sigma-Aldrich (St. Louis, MO, USA) and Merck (Peruana S.A., Lima, Peru), and were used as supplied, without further purification. A detailed list of the reagents, their applications, purity, and suppliers is presented below:

- Methanol (CH_3_OH) used for extraction of bioactive compounds (HPLC grade—Merck; Sigma-Aldrich)
- Acetic acid (CH_3_COOH) used for extraction and mobile phase preparation was at analytical grade (Merck)
- Folin–Ciocalteu used as a reagent for total phenolic content and phenolic acid assays was at analytical grade (Sigma-Aldrich)
- Sodium carbonate (Na_2_CO_3_, 7.5%) needed for color development in TPC assay was at analytical grade (Merck)
- Gallic acid used as a calibration standard for total phenolic content (≥99% purity, HPLC, Sigma-Aldrich)
- Quercetin used as a calibration standard for total flavonoids content (≥95% purity, HPLC, Sigma-Aldrich)
- Caffeic acid used as a calibration standard for phenolic acids content (≥98% purity, Sigma-Aldrich)
- β-Carotene used as a standard for carotenoid quantification (≥95%, purity, Sigma-Aldrich)
- Trolox as a calibration standard for antioxidant assays (≥97%, purity, Sigma-Aldrich)
- DPPH (2,2-diphenyl-1-picrylhydrazyl) free radical scavenging assay was ≥95% purity, (Sigma-Aldrich)
- Tannic acid used as a calibration standard for tannins (≥99%, purity, Sigma-Aldrich)
- n-Butanol used for extraction of alkaloids and saponins was analytical grade (Merck)
- Vanillin (5% in acetic acid) used as color reagent for saponin determination (analytical grade, Sigma-Aldrich)
- Diethyl ether was used for extraction of saponins (analytical grade, Merck)
- All reagents and solvents were used as supplied without further purification.

### 2.3. Preparation of Flower Extracts

To determine the content of bioactive compounds, methanolic extracts were prepared as follows: 1 g of lyophilized flower sample was mixed with 100 mL of 80% methanol. The mixture was stirred on a magnetic stirrer at room temperature for 5 h and centrifuged at 6000 rpm, 4 °C for 10 min (Eppendorf 5430R centrifuge, Eppendorf, Hamburg, Germany). The resulting supernatants were stored at −4 °C until further analyses.

The extraction yield was calculated as the ratio of the mass of the dried methanolic extract obtained to the initial dry weight of the sample, expressed as a percentage. The extraction efficiency was determined for each species to ensure comparable recovery of bioactive compounds from the analyzed flowers. The extraction yields of methanolic extracts ranged from approximately 18.4% (*C. pepo*) to 22.7% (*C. maxima*), indicating consistent extraction efficiency across the five *Cucurbita* species.

Extraction yields were comparable across all species, confirming uniform efficiency of the applied methanolic extraction procedure.

### 2.4. Determination of Total Phenolics Content

The assay was conducted according to the method of Singleton et al. [20] with slight modifications. Briefly, 0.5 mL of methanolic flower extract was mixed with 2.5 mL of diluted Folin–Ciocalteu reagent (1:10). The mixture was incubated for 10 min at room temperature, followed by the addition of 2.0 mL of 7.5% Na_2_CO_3_ solution. After thorough mixing, the samples were incubated in darkness for 30 min at room temperature. Absorbance was measured at 760 nm using a UV-2600i plus spectrophotometer (Shimadzu, Kyoto, Japan). Gallic acid was used as a calibration standard (0–200 mg mL^−1^), and results were expressed as mg gallic acid equivalents (GAE) per g dry weight (d.w.).

To minimize potential matrix effects and pigment-related interferences, all spectrophotometric measurements were performed using appropriately diluted extracts and read against reagent blanks. Additionally, absorbance was measured at specific wavelengths characteristic for each compound class to reduce spectral overlap with carotenoids and anthocyanins naturally present in the flower matrix.

### 2.5. Determination of Total Flavonoid Content (TFC)

The total flavonoid content, representing the overall class of flavonoid compounds including flavonols, flavones, and related derivatives, was determined using the method of Chang et al. [21], with slight modifications. To a test tube, 0.5 mL of methanolic extract was added along with 1.5 mL of 80% methanol, 0.1 mL of 10% AlCl_3_, 0.1 mL of 1 M potassium acetate, and 2.8 mL of distilled water. The mixture was incubated for 30 min at room temperature in darkness. Absorbance was measured at 415 nm against a blank sample (methanol instead of extract). A quercetin calibration curve (0–100 mg mL^−1^) was used, and results were expressed as mg quercetin equivalents (QE) per g d.w.

### 2.6. Determination of Total Phenolic Acids

The total phenolic acid content was measured spectrophotometrically using the Folin–Ciocalteu reagent, following Djeridane et al. [22] with modifications. Caffeic acid was used as the calibration standard, and results were expressed as mg caffeic acid equivalents (CAF eq) per 100 g d.w.

Approximately 0.5 g of dried flower powder was extracted with 10 mL of 80% methanol (*v*/*v*) for 60 min in darkness on a horizontal shaker (150 rpm). After centrifugation (5000 rpm, 10 min), the supernatant was collected. To 0.1 mL of extract, 0.5 mL of diluted Folin–Ciocalteu reagent (1:10) and, after 3 min, 1.5 mL of 7.5% Na_2_CO_3_ were added. The mixture was incubated for 30 min at 25 °C in darkness, and absorbance was measured at 765 nm using a UV-2600i plus spectrophotometer (Shimadzu, Kyoto, Japan).

### 2.7. Determination of Anthocyanins in Dried Pumpkin Flowers

Anthocyanin content was determined following the method of Giusti and Wrolstad [23], with slight modifications. A 0.5 g sample of dried flowers was extracted with 25 mL of methanol containing 0.1% HCl (85:15, *v*/*v*). Ultrasonic extraction was performed (35 kHz) for 30 min at room temperature (~22 °C) in darkness. Samples were centrifuged at 4000 rpm, 10 °C, for 10 min, and the supernatant was collected. Two aliquots of 1 mL extract were prepared: one was mixed with 4 mL of pH 1.0 buffer, the other with 4 mL of pH 4.5 buffer. After 15 min incubation in darkness at room temperature, absorbance was measured at 520 nm and 700 nm against buffer blanks. Anthocyanin concentration was calculated (mg cyanidin-3-glucoside equivalents per 100 g d.w.).

### 2.8. Determination of Total Carotenoids Content

The total carotenoids content was determined spectrophotometrically based on extraction with organic solvents and absorbance measurement at 450 nm, according to the procedure described by Lichtenthaler and Wellburn [24], adapted for dried plant material. Results were expressed as mg of total carotenoids per 100 g of dry weight (mg 100 g^−1^ d.w.). For extraction, 0.5 g of lyophilized pumpkin flower powder was mixed with 10 mL of acetone:hexane (1:3, *v*/*v*). The mixture was homogenized in the dark for 30 min and then centrifuged at 5.000 rpm for 10 min. All procedures were conducted under limited light conditions to minimize carotenoid degradation.

The absorbance of the extract was measured at 450 nm in a 1 cm quartz cuvette. If necessary, the extract was diluted to obtain absorbance values within the measurement range (0.2–1.0). Acetone:hexane (1:3) was used as the reference solvent.

(1) $$Totalc\;arotenoids\;(mg/100g)=\frac{A\times V\times 10^{4}}{A_{1\%}^{1\;cm}\times m}$$

where *A*—absorbance at 450 nm, *V*—extract volume (mL), $A_{1\%}^{1cm}$—extinction coefficient for carotenoids in a given solvent (β-carotene in ether = 2592), *m*—sample weight (mg).

### 2.9. Identification of Phenolic Acids by HPLC-DAD-ESI-MS

The identification and characterization of the particular phenolic compounds present in the methanolic extracts of pumpkin flower samples were carried out using high-performance liquid chromatography coupled with diode array detection (HPLC-DAD) and liquid chromatography–mass spectrometry (LC-MS/MS). Chromatographic separation was performed on a ZORBAX Eclipse Plus C18 column (250 × 4.6 mm, 5 µm; Agilent, Santa Clara, CA, USA) using a gradient elution of solvent A (0.1% formic acid in water) and solvent B (methanol) at a flow rate of 1.0 mL min^−1^. The gradient program was as follows: 0–5 min, 5% B; 5–25 min, 5–30% B; 25–40 min, 30–60% B; 40–50 min, 60–100% B; 50–60 min, 100% B; and 60–70 min, re-equilibration to 5% B. Detection wavelengths were set at 280 nm (hydroxybenzoic acids), 320 nm (hydroxycinnamic acids), and 360 nm (flavonoids). Mass spectrometric analyses were performed in negative ionization mode (ESI−) using a Thermo Scientific LCQ Fleet LC-MS system (San Jose, CA, USA). The mass range was scanned from *m*/*z* 100 to 1000. Identification was confirmed by comparing retention times, UV–Vis absorption maxima, and MS/MS fragmentation patterns with those of authentic standards (Sigma-Aldrich, St. Louis, MO, USA) and literature data [20,22,25,26].

The present HPLC-DAD-ESI-MS analysis focused on the identification of phenolic acids, which represent the dominant class of phenolic compounds in pumpkin flowers. Flavonoids were not specifically identified or quantified chromatographically in this study; however, their total content was determined spectrophotometrically (Section 2.5). Considering their well-recognized contribution to antioxidant activity, future research will extend the chromatographic analysis to include detailed profiling of flavonoids and carotenoids in *Cucurbita* flower extracts.

The HPLC-DAD-ESI-MS analysis allowed unambiguous identification of the main phenolic acids in the flower extracts. The detected compounds included gallic, protocatechuic, p-hydroxybenzoic, caffeic, ferulic, p-coumaric, and vanillic acids. Their retention times, UV–Vis maxima, and MS fragmentation ions confirming the reliability of the identification and enabling assessment of species-specific variations discussed in Section 3.2.

### 2.10. Determination of Antioxidant Properties

The antioxidant activity of pumpkin flower extracts was assessed using three spectrophotometric methods targeting different reactive species. The DPPH assay evaluates the scavenging capacity toward the stable nitrogen-centered radical DPPH^•^, representing the ability to neutralize free radicals through electron or hydrogen atom donation. The FRAP and CUPRAC assays, in contrast, measure the reducing potential of antioxidants toward ferric (Fe^3+^) and cupric (Cu^2+^) ions, respectively, reflecting the overall electron-donating (redox) capacity rather than scavenging of specific radicals. The hydroxyl radical scavenging assay (^•^OH) based on deoxyribose degradation was used to determine the ability of extracts to neutralize highly reactive oxygen species.

#### 2.10.1. Determination of Reducing Capacity Using the FRAP Assay

The reducing capacity of methanolic extracts from pumpkin flowers was determined using the FRAP method according to Benzie et al. [27], with minor modifications. Briefly, 3.3 mL of acetate buffer (pH 3.6) was mixed with 0.33 mL of 20 mM FeCl_3_ and 0.33 mL of 10 mM TPTZ solution. After incubation at 37 °C for 5 min, 0.33 mL of the methanolic extract was added. The mixture was incubated for an additional 15 min at 37 °C, and absorbance was measured at 450 nm using a UV–Vis spectrophotometer (UV-2600i Plus, Shimadzu, Japan). A calibration curve was prepared in the range of 200–1200 μM using an aqueous solution of Fe(III) sulfate as the standard. The reducing capacity was expressed as μmol Fe(II) per g of dry plant material (μmol Fe(II) g^−1^ d.w.).

#### 2.10.2. Determination of Reducing Capacity Using the CUPRAC Assay

The reducing capacity of the pumpkin flower extracts was determined using the CUPRAC method according to Apak et al. [28], with minor modifications. Briefly, 0.5 mL of methanolic extract was mixed with 1 mL of 10 mM CuCl_2_, 1 mL of 7.5 mM neocuproine (2,9-dimethyl-1,10-phenanthroline), 1 mL of ammonium acetate buffer (pH 7.0), and 0.5 mL of deionized water. Absorbance was measured at 450 nm using a UV–Vis spectrophotometer (UV-2600i Plus, Shimadzu, Japan). A calibration curve was prepared with Trolox standard solutions in the range of 0–0.009 μM. The reducing capacity was expressed as μmol Trolox equivalents (TE) per g of dry plant material (μmol TE g^−1^ d.w.).

#### 2.10.3. Determination of Free Radical Scavenging Activity Using the DPPH Assay

The DPPH free radical scavenging activity was determined according to the method of Brand-Williams et al. [29], with minor modifications. Briefly, 1 mL of 0.1 mM DPPH solution and 1 mL of methanolic extract from the pumpkin flowers were mixed in a test tube. The mixture was thoroughly mixed and incubated in the dark at room temperature for 30 min. Absorbance was measured at 517 nm against a blank (DPPH + methanol, without extract) using a UV–Vis spectrophotometer (UV-2600i Plus, Shimadzu, Japan). A calibration curve was prepared using Trolox standard solutions in the range of 100–1000 μM. The DPPH scavenging activity was expressed as μM Trolox equivalents per g of dry weight (μM Trolox g^−1^ d.w.).

#### 2.10.4. Determination of Hydroxyl Radical Scavenging Activity Using the Deoxyribose Degradation Assay

Hydroxyl radical scavenging activity was determined according to the method described by Halliwell et al. [30], with minor modifications. Briefly, 0.4 mL of methanolic extract from the pumpkin flowers was mixed with 0.2 mL of 28 mM deoxyribose solution. Subsequently, 1 mL of phosphate buffer (pH 7.4), 0.2 mL of aqueous FeCl_3_ solution, 0.2 mL of 1 mM EDTA solution, 0.2 mL of 1 mM H_2_O_2_ solution, and 0.2 mL of 1% ascorbic acid solution were added, and the mixture was incubated at 37 °C for 60 min. After incubation, 1 mL of 1% thiobarbituric acid solution and 1 mL of 0.05 M trichloroacetic acid solution were added. The mixture was incubated for 15 min in a boiling water bath, and absorbance was measured at 530 nm. A calibration curve was prepared in the range of 0–400 μM using quercetin as a standard. Results were expressed as μM quercetin equivalents (QE) per 1 g of dry plant material. All spectrophotometric analyses were performed using a UV–Vis spectrophotometer (UV-2600i Plus, Shimadzu, Japan).

For benchmarking purposes, Trolox, gallic acid, and quercetin standards were used as reference antioxidants in calibration curves for DPPH, FRAP, CUPRAC, and hydroxyl radical scavenging assays. These reference compounds, with well-documented antioxidant potency, served as positive controls for assessing the relative activity of the pumpkin flower extracts.

To ensure correct assessment of antioxidant activity, all assays were calibrated against standard antioxidant substances: Trolox (for DPPH and CUPRAC), Fe(II) sulfate (for FRAP), and quercetin (for hydroxyl radical scavenging). Results were expressed as Trolox equivalents (TE), quercetin equivalents (QE), or Fe(II) equivalents per gram of dry weight (d.w.), allowing direct comparison with literature data on known antioxidant systems.

### 2.11. Determination of Antinutritional Compounds

#### 2.11.1. Determination of Tannins Content by Spectrophotometry

The tannins content in flowers of five pumpkin species was determined according to the method described by Saxena and Chagti [31], with minor modifications, using the Folin–Denis reagent. Briefly, 0.5 g of lyophilized pumpkin flower sample was placed in a 250 mL conical flask and mixed with 75 mL of distilled water. The flask was gently heated and then boiled for 30 min. After cooling, the mixture was centrifuged at 10,000 rpm for 10 min, and the supernatant was transferred to a 100 mL volumetric flask and brought to volume with distilled water. Next, 1 mL of the sample extract was transferred to a 100 mL volumetric flask containing 75 mL of distilled water, mixed with 5 mL of Folin–Denis reagent and 10 mL of sodium carbonate solution, and diluted to 100 mL with distilled water. The samples were incubated at 25 °C for 30 min, and absorbance was measured at 700 nm using a UV–Vis spectrophotometer (UV-2600i Plus, Shimadzu, Japan). Tannins concentration was calculated based on a standard curve prepared with tannic acid.

% Tannins (mg/100 g) = (A × C × R)/(Aw × M × 100)(2)

where A—absorbance of the sample, Aw—absorbance of the standard tannic acid solution, C—concentration of the standard tannic acid solution (mg mL^−1^), R—dilution factor, M—mass of the sample (mg).

#### 2.11.2. Determination of Phytates Content by Titration Method

Phytates were determined by titration according to the method described by Lolas and Markakis [32], with minor modifications. Two grams of lyophilized pumpkin flower sample were placed in a 250 mL conical flask and soaked with 100 mL of 2% HCl for 3 h. After incubation, the sample was filtered through Whatman No. 1 filter paper. A 50 mL aliquot of the filtrate was mixed with 10 mL of distilled water and titrated against a standard FeCl_3_ solution containing 0.00195 g Fe mL^−1^ until a brown-yellow color persisted for 5 min. The percentage of phytic acid was then calculated according to the following formula:

% Phytic acid = y × 1.19 × 100(3)

where y = titre value × 0.00195 g.

#### 2.11.3. Determination of Oxalates Content

The oxalates content in the analyzed samples was determined according to the method described by Chinma and Igyor [33], with minor modifications. One gram of lyophilized pumpkin flower sample was mixed with 75 mL of 3 M H_2_SO_4_ and stirred for 1 h on a magnetic stirrer (SBS-MR-1500/H, Steinberg System, Berlin, Germany). The mixture was then filtered through Whatman No. 1 filter paper. A 25 mL aliquot of the filtrate was titrated hot against 0.05 M KMnO_4_ until a faint pink color persisted for at least 30 s. The oxalate content was calculated assuming that 1 mL of 0.05 M KMnO_4_ corresponds to 2.2 mg of oxalates.

#### 2.11.4. Determination of Alkaloids Content

The total alkaloid content was determined by titration according to the method described by Rastogi and Mehrotra [34], with minor modifications. Five grams of lyophilized pumpkin flower sample were mixed with 20 mL of n-butanol and vigorously shaken. The mixture was transferred to a bottle and kept at room temperature for 12 h. After incubation, the mixture was centrifuged at 10,000 rpm for 10 min, and the obtained supernatant was adjusted to 50 mL with n-butanol.

A 10 mL portion of the supernatant was transferred to a 100 mL separating funnel together with 10 mL of 0.1 N HCl, and the mixture was shaken thoroughly for 2–3 min. The lower aqueous layer containing the neutralized alkaloids was collected into a beaker, and 2–3 drops of methyl red indicator were added, producing a slightly reddish solution. The sample was then titrated with 0.1 N NaOH until the color changed from red to light yellow, persisting for at least 30 s. Each sample was titrated in triplicate. The total alkaloid content was calculated assuming that 1 mL of 0.1 N HCl is equivalent to 0.0162 g of alkaloid.

#### 2.11.5. Determination of Saponins Content

The total saponins content was determined spectrophotometrically according to the procedure described by Obadoni and Ochuko [35], with minor modifications. Two grams of lyophilized pumpkin flower sample were mixed with 100 mL of 20% aqueous ethanol and incubated in a water bath (WE series, Laboplay, Bytom, Poland) at 55 °C for 4 h, with intermittent shaking. The mixture was filtered and re-extracted with 200 mL of 20% ethanol, after which the combined extract was reduced to a volume of 40 mL. The filtrate was transferred to a 250 mL separating funnel, to which 20 mL of diethyl ether was added, followed by vigorous shaking. The aqueous layer was collected and extracted three times with 30 mL of n-butanol. The n-butanol extract was washed three times with 10 mL of 5% NaCl solution and then evaporated to dryness. The residue was dried in an oven at 40 °C to constant weight.

For the colorimetric assay, 0.2 mL of vanillin-acetic acid reagent (5% *w*/*v*) and 0.8 mL of perchloric acid were added to half of the n-butanol extract, and the mixture was heated at 70 °C for 15 min. The reaction mixture was cooled in an ice bath for 1 min, followed by the addition of 5.0 mL of ice-cold acetic acid. The absorbance of the samples was measured at 550 nm using a UV–Vis spectrophotometer (UV-2600i Plus, Shimadzu, Japan).

### 2.12. Statistical Analysis

All analyses were performed in triplicate. All measurements were performed in triplicate (*n* = 3), and the results are expressed as mean ± standard deviation (SD). Differences between mean values were evaluated using one-way ANOVA followed by Tukey’s post hoc test (*α* = 0.05). The obtained results were subjected to statistical analysis using the Statistica 8.0 software package. One-way analysis of variance (ANOVA) was performed, and the significance of differences between means was determined using Tukey’s test (α = 0.05). Comparative analysis among the five *Cucurbita* species was performed for each parameter (TPC, TFC, TPAC, carotenoids, and anthocyanins) using one-way ANOVA, and the results were expressed as mean ± SD. Differences between means were considered significant at *p* < 0.05 according to Tukey’s test.

In addition to ANOVA, Pearson’s correlation coefficients were calculated to determine the relationships between the concentrations of bioactive compounds (TPC, TFC, TPAC, carotenoids, and anthocyanins) and antioxidant activity indices (DPPH, FRAP, CUPRAC, and ^•^OH).

For consistency and comparability, all concentrations of bioactive compounds were expressed in milligrams of equivalent per gram of dry weight (mg E g^−1^ d.w.), where E refers to the corresponding standard (GAE for total phenolics, QE for flavonoids, CAF eq for phenolic acids, C3G eq for anthocyanins, and β-carotene eq for carotenoids). All values were recalculated accordingly and are reported uniformly throughout the manuscript.

## 3. Results and Discussion

### 3.1. Content of Polyphenols, Flavonoids, Phenolic Acids, Carotenoids and Anthocyanins

Extraction yields of methanolic extracts did not differ significantly among species, ensuring that variations in the levels of bioactive compounds reflected true interspecific differences rather than extraction efficiency effects.

#### 3.1.1. Total Polyphenols Content

The mean total polyphenol content in the analyzed pumpkin flowers was 40.94 mg GAE g^−1^ d.w. (Table 1). The values varied among pumpkin species, ranging from 34.20 mg GAE g^−1^ d.w. in summer squash to 47.29 mg GAE g^−1^ d.w. in giant pumpkin. These findings are consistent with those reported by Socha et al. [36]. In contrast, Ghosh and Rana [18] reported polyphenol contents of 8.09 µg mL^−1^ GAE in aqueous extracts and 17.39 µg mL^−1^ GAE in methanolic extracts of pumpkin flowers. Similarly, Kandylis [37] observed polyphenol levels of 13 mg GAE g^−1^ d.w. in summer squash. Bieżanowska-Kopeć et al. [17], investigating fresh pumpkin flowers, reported total polyphenol contents of 133.26 mg CAE 100 g^−1^ (chlorogenic acid equivalents). In their study, the highest value was found in the Atlantic Giant variety (182.07 mg CAE 100 g^−1^), while the lowest was recorded in the Bambino variety (104.36 mg CAE 100 g^−1^). Toro-Velez et al. [38] also demonstrated high polyphenol levels in fresh pumpkin flowers, with contents calculated as gallic acid equivalents reaching approximately 320–340 mg GAE 100 g^−1^ FW. Comparable variability was observed by Zhou et al. [39], whose results align with our findings. They reported marked species-related differences in polyphenol levels, with 50.17 mg GAE 100 g^−1^ FW in summer squash, and much higher concentrations in giant pumpkin and butternut squash (436.16 and 453.72 mg GAE 100 g^−1^ FW, respectively). It should be noted that the high pigmentation of certain *Cucurbita* species (particularly *C. ficifolia* and *C. maxima*) could potentially interfere with colorimetric assays due to overlapping absorption spectra of carotenoids and anthocyanins. However, careful wavelength selection, reagent blank correction, and sample dilution minimized such matrix effects, ensuring reliable quantification of phenolic compounds.

The results presented in Table 1 show the mean values ± standard deviation of total phenolic content (TPC), total flavonoid content (TFC), total phenolic acid content (TPAC), total carotenoids, and anthocyanins for flowers of the five *Cucurbita* species. Statistical differences between the mean values were evaluated using one-way ANOVA followed by Tukey’s post hoc test (α = 0.05).

#### 3.1.2. Total Carotenoids Content

Carotenoids belong to the group of plant-derived antioxidants that function both as pigments and as bioactive compounds with antioxidant properties. Di Lorenzo et al. [40] and Kawata et al. [41] confirmed in their studies that pumpkin flowers are a rich source of carotenoids and may exert anti-inflammatory effects. In the present study, the mean carotenoid content in the dry matter of pumpkin flowers was 39.93 mg 100 g^−1^, with considerable variation among species (Table 1). According to Bieżanowska-Kopeć et al. [17], the highest carotenoid concentrations are typically observed in flowers with an intensely orange coloration. Our findings are consistent with those observations, as the flowers of fig-leaf gourd, characterized by a deep orange color, contained the highest total carotenoid content (48.27 mg 100 g^−1^). In contrast, the flowers of cushaw squash exhibited the lowest concentration (31.35 mg 100 g^−1^). Similar results were reported by Murkovic et al. [42] for light orange–yellow flowers, in which total carotenoid content ranged from 57 to 2760 μg g^−1^ fresh weight. Numerous studies have documented substantial diversity in carotenoid profiles across different pumpkin varieties [43]. Both the total carotenoid content and the composition of individual carotenoids are highly variable and depend on factors such as cultivar and species, soil and climatic conditions, agronomic practices, plant part (pulp, peel, or seeds), as well as extraction protocols [17,43]. Interestingly, Toro-Velez et al. [38] reported that the β-carotene content in pumpkin flowers increased during storage, compared to fresh flowers, in which it averaged 10–14 mg β-carotene 100 g^−1^ fresh weight.

#### 3.1.3. Flavonoids

Flavonoids, as compounds with a broad spectrum of biological activities, are currently the subject of intensive research regarding their role in the prevention of chronic diseases. Evidence from in vitro, in vivo, and clinical studies has demonstrated their promising antidiabetic, anti-inflammatory, antibacterial, antioxidant, antiviral, cytotoxic, and lipid-lowering effects [44,45,46]. In this context, Pumpkin species with high total flavonoid levels, particularly those rich in flavonols such as quercetin derivatives, may be of particular interest for the development of functional foods or phytotherapeutics. In the present study, the mean total flavonoid content in pumpkin flowers was 18.77 mg QE g^−1^ d.w. The values varied among species, ranging from 15.70 mg QE g^−1^ d.w. in fig-leaf gourd to 21.32 mg QE g^−1^ d.w. in giant pumpkin. The assay used quantification relative to quercetin, which belongs to the flavonol subclass; however, the obtained TFC values represent the total pool of flavonoid compounds rather than flavonols exclusively. Flavonoids encompass several structural subclasses, including flavonols, flavones, flavanones, and anthocyanidins, which collectively contribute to the antioxidant potential of plant tissues. The highest content was observed in the giant pumpkin flowers, which was statistically significantly higher compared to the other species, indicating the considerable potential of giant pumpkin as a source of flavonoids. The flowers of cushaw squash also contained relatively high flavonol levels (20.40 mg QE g^−1^ d.w.), suggesting that this species may likewise serve as a valuable source of flavonoids. Butternut squash flowers contained a moderate amount (19.27 mg QE g^−1^ d.w.), followed by summer squash (17.16 mg QE g^−1^ d.w.), whereas fig-leaf gourd flowers exhibited the lowest flavonoid content (15.70 mg QE g^−1^ d.w.).

These results confirm that pumpkin flowers are a source of flavonoids, and that their content is species-dependent. Variability in flavonol levels may arise not only from genetic factors but also from environmental conditions such as cultivation practices, sunlight exposure, mineral availability, and oxidative stress in plants [47,48]. In summary, giant pumpkin and cushaw squash flowers exhibit the greatest potential for flavonol accumulation, whereas fig-leaf gourd and summer squash accumulate considerably lower levels. These differences likely reflect distinct genetic and metabolic characteristics among species, which should be considered when selecting plant material for further research or practical applications.

#### 3.1.4. Total Phenolic Acids

The mean content of phenolic acids in the analyzed samples was 2.17 mg CAE g^−1^ d.w., with values ranging from 2.11 to 2.29 mg CAE g^−1^ d.w. The flowers of fig-leaf gourd exhibited the highest phenolic acid content (2.29 mg CAE g^−1^ d.w.), which was statistically significantly higher compared to the other species (*p* < 0.05). The remaining species—giant pumpkin, summer squash, butternut squash, and cushaw squash—showed comparable phenolic acid levels (2.13, 2.11, 2.17, and 2.16 mg CAE g^−1^ d.w., respectively). Statistical analysis confirmed that these species form a homogeneous group in this regard. These findings confirm a moderate yet significant contribution of phenolic acids to the biochemical composition of pumpkin flowers, complementing previous studies that demonstrated species of the genus *Cucurbita* as sources of phenolic compounds with antioxidant properties [1,2,45,46]. Particularly noteworthy is the distinct profile of fig-leaf gourd flowers, which makes this species a valuable raw material for further research into potential health-promoting or industrial applications (e.g., as a source for the extraction of natural antioxidants). Although both assays—the total phenolic content (TPC) and total phenolic acid content (TPAC)—employ the Folin–Ciocalteu reagent, they measure different subsets of phenolic compounds and rely on distinct calibration standards (gallic acid and caffeic acid, respectively). The observed differences between TPC and TPAC values arise primarily from the broader range of phenolic constituents detected in the TPC assay, which also responds to flavonoids, tannins, and other reducing substances, whereas the TPAC assay selectively quantifies phenolic acids only. Additionally, differences in extraction time, solvent polarity, and sample dilution may influence matrix interactions with the Folin–Ciocalteu reagent, further explaining the observed quantitative discrepancies between the two determinations.

The highest concentration of anthocyanins was found in butternut squash flowers (12.18 mg cyn-3-glu eq 100 g^−1^), whereas the lowest was observed in fig-leaf gourd flowers (8.48 mg cyn-3-glu eq 100 g^−1^). The mean content across all analyzed samples was 10.83 mg 100 g^−1^. The observed differences may result from differential expression of genes involved in anthocyanin biosynthesis, which is strongly influenced by both genetic and environmental factors [49]. These results are consistent with previous reports indicating that different parts of *Cucurbita* plants, including flowers and fruits, may contain considerable amounts of anthocyanins and other phenolic compounds [50,51].

It is also important to emphasize that although summer squash and fig-leaf gourd contained lower levels of anthocyanins, their antioxidant potential may remain high due to the presence of other bioactive compounds, such as carotenoids and flavonoids [52]. Anthocyanin content in flowers may also hold technological and esthetic relevance, for instance in the context of utilizing plant extracts as natural colorants [53]. Overall, the results of the present study highlight the potential of pumpkin flowers, particularly those of butternut squash and giant pumpkin, as sources of natural anthocyanins with health-promoting and practical applications.

### 3.2. Content of Individual Phenolic Acids

Key phenolic acids were identified and confirmed using HPLC-DAD-ESI-MS based on retention times, UV–Vis absorption maxima, and MS/MS fragmentation patterns. Seven major phenolic acids—gallic, protocatechuic, p-hydroxybenzoic, caffeic, ferulic, p-coumaric, and vanillic acids—were detected across all species. Their molecular characteristics (λmax, [M–H]^−^, and fragment ions) are summarized in Table 2. Species-specific variations in the quantitative distribution of these compounds are shown in Table 3, indicating clear interspecific differences in phenolic acid profiles.

All analyzed pumpkin species contained considerable amounts of six main phenolic acids: ferulic, gallic, p-hydroxybenzoic, caffeic, p-coumaric, protocatechuic, and vanillic acids (Table 2). Although only phenolic acids were identified by HPLC-DAD-ESI-MS, the presence of flavonoids in pumpkin flower extracts was confirmed by spectrophotometric assays (Table 1). These compounds, along with carotenoids, likely contribute synergistically to the observed antioxidant activity.

Differences between species were pronounced, suggesting variations in metabolic activity and antioxidant potential among the studied species. The dominant phenolic compound in all flowers was p-coumaric acid, with concentrations ranging from 124.40 mg 100 g^−1^ in cushaw squash to 151.51 mg 100 g^−1^ in fig-leaf gourd. These results indicate that this acid plays a key role in the phenolic profile of pumpkin flowers, consistent with previous reports highlighting its significance in plant defense against oxidative stress [54]. The second most abundant compound was caffeic acid, with relatively similar levels across species, ranging from 25.61 mg 100 g^−1^ (butternut squash) to 28.41 mg 100 g^−1^ (giant pumpkin). These compounds are well-known for their strong antioxidant and anti-inflammatory properties [55], which enhance the functional value of the flowers (Table 3).

Interestingly, the highest gallic acid content was found in fig-leaf gourd flowers (23.19 mg 100 g^−1^), clearly exceeding values observed in the other species (18.41–20.17 mg 100 g^−1^). Gallic acid is a potent antioxidant capable of scavenging free radicals and chelating metal ions [56], suggesting a higher protective potential of this species despite generally lower concentrations of other phenolic acids.

Ferulic acid content ranged from 17.27 to 19.60 mg 100 g^−1^, being higher in butternut squash and giant pumpkin flowers compared to the other species. Summer squash exhibited an intermediate level (18.72 mg 100 g^−1^), while fig-leaf gourd and cushaw squash had the lowest concentrations. The presence of ferulic acid in pumpkin flowers complements previous data on *Cucurbitaceae* fruits [57], where it contributed to pigment stability and antioxidant properties.

The highest p-hydroxybenzoic acid content was found in cushaw squash flowers (16.93 mg 100 g^−1^) and the lowest in summer squash (12.77 mg 100 g^−1^). This acid possesses antibacterial activity and serves as a precursor for several phytoalexins involved in plant defense [58].

Protocatechuic acid was most abundant in fig-leaf gourd flowers (16.27 mg 100 g^−1^) and lowest in butternut squash flowers (12.22 mg 100 g^−1^). This acid exhibits hepatoprotective and cardioprotective effects [59], potentially enhancing the nutraceutical value of the raw material.

Vanillic acid was highest in fig-leaf gourd flowers (12.31 mg 100 g^−1^) and lowest in cushaw squash (6.80 mg 100 g^−1^). Despite its low overall concentration, this compound is significant due to its antioxidant and potential neuroprotective properties [60].

Pumpkin flowers, regardless of species, are a rich source of bioactive phenolic acids with potential health-promoting effects. The highest total phenolic content was found in giant pumpkin and fig-leaf gourd flowers, making them particularly valuable raw materials for functional foods and dietary supplements.

### 3.3. Antioxidant Properties

According to our study, giant pumpkin flowers exhibited a higher antioxidant potential compared to the other cultivars, which may be attributed to their specific profile of secondary metabolites, including phenolic acids (Table 4). In contrast, summer squash flowers showed the lowest antioxidant activity among the species tested, regardless of the applied assay.

The antioxidant activities observed in pumpkin flower extracts were of the same order of magnitude as those reported for other plant materials with established antioxidant properties, such as green tea (*Camellia sinensis*), rosemary (*Rosmarinus officinalis*), or grape skin (*Vitis vinifera*) [61,62,63,64,65,66]. This comparison confirms that certain *Cucurbita* species—particularly *C. maxima* and *C. moschata*—exhibit considerable antioxidant potential comparable to recognized natural antioxidants.

The in vitro antioxidant assays revealed marked interspecific variability in radical scavenging and reducing capacities among the five *Cucurbita* species. When expressed as Trolox equivalents, the DPPH and CUPRAC values of *C. maxima* and *C. argyrosperma* flowers (11.22–11.50 μmol TE g^−1^ d.w.) were comparable to those reported for several medicinal and aromatic plants with well-documented antioxidant potential, such as basil (*Ocimum basilicum*, 9.8–12.3 μmol TE g^−1^ d.w.), rosemary (*Rosmarinus officinalis*, 10.5–13.6 μmol TE g^−1^ d.w.), and green tea (*Camellia sinensis*, 14.2–16.7 μmol TE g^−1^ d.w.) [61,62,63,64,65,66]. This indicates that pumpkin flowers, particularly those of *C. maxima* and *C. argyrosperma*, exhibit strong antioxidant performance comparable to recognized natural antioxidants. Moreover, the high FRAP and ^•^OH scavenging capacities correlated positively with total phenolic (TPC) and flavonoid (TFC) contents, confirming that these bioactive compounds are the main contributors to the antioxidant mechanisms of pumpkin flower extracts. Carotenoids and anthocyanins also play a complementary role, contributing to the overall redox potential of the extracts.

The differences observed between species may result from genetic and environmental factors (climate, soil, harvest stage), as well as variations in enzymatic activity responsible for phenolic biosynthesis [67]. These findings underscore the need for further research on standardization of the raw material and its application in the food and pharmaceutical industries.

Overall, pumpkin flowers, irrespective of species, are a source of numerous bioactive compounds, including phenolic acids with potential antioxidant properties, as confirmed by assays assessing antioxidant capacity.

#### Pearson’s Correlation

To explore the relationships between the concentrations of bioactive compounds and the antioxidant capacity of the studied *Cucurbita* flowers, Pearson’s correlation analysis was conducted. Strong positive correlations (r = 0.78–0.94, *p* < 0.05) were observed between total phenolic (TPC) and flavonoid contents (TFC) and the antioxidant indices (DPPH, FRAP, CUPRAC), indicating that phenolic compounds were the primary contributors to the antioxidant potential (Table 5). Negative associations were also found between carotenoids and antioxidant activity, whereas anthocyanins showed species-dependent relationships.

### 3.4. Content of Antinutritional Compounds

In parallel with the analysis of antioxidant compounds, a concurrent assessment of key antinutritional factors—including tannins, phytates, oxalates, alkaloids, and saponins—was performed. This comparative approach across the five *Cucurbita* species enabled interspecies evaluation of these compounds and provided translational insights relevant to the culinary use and technological processing of pumpkin flowers.

#### 3.4.1. Tannins

The highest tannins concentration was observed in butternut squash flowers (2.21 mg 100 g^−1^ d.w.), which may indicate stronger antioxidant and protective properties for this species (Table 6). The lowest tannin content was recorded in cushaw squash flowers (1.78 mg 100 g^−1^ d.w.), suggesting reduced bitterness and potentially greater palatability, but also a lower protective capacity against pathogens. The remaining species exhibited tannin concentrations of 2.10 mg 100 g^−1^ (fig-leaf gourd), 1.98 mg 100 g^−1^ (summer squash), and 1.90 mg 100 g^−1^ (giant pumpkin), reflecting moderate levels that may influence their health-promoting and industrial properties to a lesser extent than in butternut squash. Singh and Kumar [67], in their study on the nutritional and phytochemical characteristics of *C. pepo*, reported that pumpkin seeds contain the highest tannin levels, reaching 28.15 mg 100 g^−1^ d.w. According to Ngozi and Nkiru [68], tannin content in plant material may vary depending on factors such as growth stage and processing methods, e.g., boiling. Cosme et al. [69] highlighted that tannins are widely present in various plant-derived foods and beverages, where they substantially affect flavor, astringency, and health benefits. They are well known for their antioxidant, anti-inflammatory, and cardioprotective properties [70], and have been associated with a reduced risk of chronic diseases, including cardiovascular disorders, cancer, and diabetes. Their bioavailability and metabolism are influenced by factors such as polymerization, solubility, and interactions with the gut microbiota [71].

#### 3.4.2. Phytates

The highest phytates content was recorded in giant pumpkin flowers (4.94 mg 100 g^−1^ d.w.), suggesting that this species has the greatest potential for phytate accumulation among those studied. Slightly lower values were found in fig-leaf gourd (4.69 mg 100 g^−1^ d.w.). Summer squash (4.50 mg 100 g^−1^ d.w.) and cushaw squash (4.34 mg 100 g^−1^ d.w.) showed moderate phytate levels, while butternut squash exhibited the lowest concentration (4.17 mg 100 g^−1^ d.w.). In a study by Halder and Khaled [72], phytate content in giant pumpkin flowers was reported at 5.07 mg 100 g^−1^ d.w.

Phytate intake in humans should not exceed 250–350 mg under a Western diet, although in some dietary patterns it may reach ≥1000 mg. Phytates are well-known antinutritional compounds with the ability to chelate metal ions such as iron, zinc, and calcium, thereby reducing the bioavailability of these essential minerals [73,74]. However, some studies also highlight their antioxidant activity, which may provide health benefits [75]. Kumar et al. [76] emphasized significant differences in phytate levels between pumpkin species and cultivars, underlining their impact on nutritional value and raw material quality. Mashitoa et al. [77] reported that technological processing methods, such as boiling, can effectively reduce phytate levels, thereby improving mineral bioavailability.

From a practical standpoint, the high phytate content in giant pumpkin and fig-leaf gourd suggests the necessity of applying reduction strategies during processing or cultivation practices. In contrast, the lower phytate content in butternut squash may make this species a more attractive raw material for nutritional applications, particularly where high mineral bioavailability is desired [73,74]. The obtained results indicate that pumpkin species differ in their potential phytate content in flowers, which is relevant for both the food industry and research on functional nutrition. The selection of appropriate species and optimization of processing methods may enhance the nutritional value of pumpkin flower–based products and improve their health functionality.

#### 3.4.3. Oxalates

The highest oxalates content was observed in flowers of summer squash (0.25 mg 100 g^−1^ d.w.), which differed significantly from the other samples, namely butternut squash and fig-leaf gourd (0.15 mg 100 g^−1^ and 0.17 mg 100 g^−1^, respectively). Relatively high oxalate levels were also recorded in giant pumpkin (0.21 mg 100 g^−1^ d.w.) and cushaw squash (0.20 mg 100 g^−1^ d.w.), with no significant differences between them. Oxalates are naturally occurring antinutritional compounds that may form insoluble calcium salts, thereby reducing calcium bioavailability and contributing to kidney stone formation. For this reason, the lower oxalate content in butternut squash and fig-leaf gourd may be considered beneficial from a nutritional and preventive health perspective, particularly for individuals at risk of nephrolithiasis [78]. Conversely, summer squash, which exhibited the highest oxalate level, may require dietary restrictions for certain patients despite its popularity and broad culinary use [79].

#### 3.4.4. Alkaloids

The highest alkaloids content was detected in fig-leaf gourd flowers (0.31 mg 100 g^−1^ d.w.), which differed significantly from the other species. Elevated alkaloids levels were also recorded in giant pumpkin and cushaw squash (0.26 mg 100 g^−1^ d.w. each), with no significant differences between them (Table 4). The lowest values were found in summer squash (0.17 mg 100 g^−1^ d.w.) and butternut squash (0.21 mg 100 g^−1^ d.w.), which differed significantly from fig-leaf gourd and, to a lesser extent, from the other species.

Alkaloids are natural compounds with strong physiological activity that, depending on concentration, may exhibit therapeutic properties. They are particularly recognized for their anesthetic, cardioprotective, and anti-inflammatory effects [80]. However, excessive consumption of certain alkaloids may lead to adverse effects such as mucosal irritation or neurotoxic action [81]. In this context, fig-leaf gourd deserves special attention due to its highest alkaloid content and potentially greater biological activity, which may be of pharmacological as well as dietary relevance. In contrast, summer squash, with the lowest alkaloids concentration, may be safer for individuals sensitive to these compounds. The alkaloids content of pumpkin flowers was also reported by Ghosh and Rana [18], who expressed their results in terms of atropine equivalents. The aqueous extract of pumpkin flowers contained 2.29 µg mL^−1^, while the methanolic extract contained 1.76 µg mL^−1^.

#### 3.4.5. Saponins

Saponins are plant secondary metabolites with surface-active properties, known for their bitter taste and broad spectrum of biological activities, ranging from immunostimulatory to anticancer and antifungal effects [82,83,84,85]. Nevertheless, excessive intake may lead to erythrocyte hemolysis or impair the absorption of certain nutrients [78,79].

In the analyzed samples, the highest saponin content was observed in summer squash (57.67 mg 100 g^−1^ d.w.) and giant pumpkin (54.67 mg 100 g^−1^ d.w.), which differed significantly from fig-leaf gourd and butternut squash. Cushaw squash showed an intermediate value (49.67 mg 100 g^−1^ d.w.), while the lowest concentrations were recorded in fig-leaf gourd (41.33 mg 100 g^−1^ d.w.) and butternut squash (44.67 mg 100 g^−1^ d.w.).

From a nutritional perspective, species whose flowers contain moderate levels of saponins—such as cushaw squash and butternut squash—may combine beneficial biological effects with a lower risk of adverse outcomes. Conversely, the high saponin content in summer squash and giant pumpkin may be advantageous for functional or pharmacological applications but requires caution in dietary use, particularly for individuals sensitive to saponin intake. According to the available literature, there are no previous studies reporting the saponin content of pumpkin flowers.

## 4. Conclusions

The conducted study demonstrated that edible pumpkin flowers represent a valuable source of bioactive compounds with antioxidant activity. The chemical composition analysis revealed significant differences in the content of bioactive compounds, including polyphenols, flavonols, phenolic acids, and carotenes. The observed antioxidant activity was primarily associated with the total flavonoid pool, including flavonol-type compounds such as quercetin derivatives. Among the studied species, the flowers of summer squash and butternut squash exhibited particularly favorable biochemical profiles, showing the highest antioxidant activity in DPPH and ABTS assays.

The analysis of antinutritional compounds such as tannins, phytates, oxalates, alkaloids, and saponins in the flowers of five pumpkin species indicated differences that may be of practical importance for their culinary use. Tannins, identified in concentrations ranging from 1.78 mg g^−1^ in cushaw squash to 2.21 mg g^−1^ in butternut squash, may contribute to a bitter taste and reduce protein digestibility. However, the relatively low tannin content in cushaw squash and summer squash may favor a more neutral taste and better consumer acceptance. Phytates, known for their ability to bind minerals and reduce their bioavailability, were present in concentrations from 4.17 mg g^−1^ in butternut squash to 4.94 mg g^−1^ in giant pumpkin. Despite their occurrence, the levels were relatively low, suggesting that they are unlikely to pose a major barrier to the culinary application of pumpkin flowers, particularly after appropriate thermal processing or fermentation. Oxalates were detected at the lowest concentrations (0.15–0.25 mg g^−1^), with the lowest content recorded in fig-leaf gourd (0.15 mg g^−1^). Such low oxalate levels are advantageous as they minimize the risk of forming insoluble calcium salts, which is relevant for individuals predisposed to kidney stone formation. Alkaloids, another important group of compounds, were identified in the range of 0.17 mg g^−1^ (summer squash) to 0.31 mg g^−1^ (fig-leaf gourd). The lowest alkaloid content in summer squash suggests that flowers of this species may be safer for direct consumption, although overall alkaloid levels were low and unlikely to pose a risk under moderate intake. Saponins, known for their foaming properties and impact on taste, showed the greatest variation, ranging from 41.33 mg g^−1^ in fig-leaf gourd to 57.67 mg g^−1^ in summer squash. Higher saponin content in summer squash flowers may impart a slightly bitter flavor and influence food texture, which should be considered when designing culinary formulations.

Overall, the results confirm the potential of pumpkin flowers as natural ingredients for functional foods. They not only serve as an attractive culinary addition but may also contribute to the prevention of civilization-related diseases associated with oxidative stress. Furthermore, the obtained data provide a valuable foundation for future research on the application of pumpkin flowers in the food, nutraceutical, and pharmaceutical industries. The concurrent evaluation of antinutritional compounds provided valuable comparative data among species, which may guide future recommendations for the culinary and technological processing of pumpkin flowers.

Correlation further confirmed that phenolic and flavonoid contents were the key determinants of antioxidant activity in the examined pumpkin flowers.

Further studies are planned to include chromatographic identification and quantification of flavonoids and carotenoids to more comprehensively characterize the compounds responsible for the antioxidant activity of pumpkin flowers.

The present study comprehensively evaluated the bioactive composition and antioxidant potential of flowers from five *Cucurbita* species—*C. maxima*, *C. moschata*, *C. pepo*, *C. ficifolia*, and *C. argyrosperma*. The comparative dataset, based on statistically analyzed mean values, revealed significant interspecific variability in the levels of phenolic acids, flavonoids, carotenoids, and anthocyanins.

The HPLC-DAD-ESI-MS analysis enabled the identification of key phenolic acids, while total phenolic (TPC), flavonoid (TFC), and phenolic acid (TPAC) contents were determined spectrophotometrically. Among the studied species, *C. maxima* and *C. moschata* showed the highest accumulation of phenolic compounds and exhibited the strongest antioxidant activity in all assays (DPPH, FRAP, CUPRAC, ^•^OH), confirming the central role of phenolics in redox mechanisms.

Correlation confirmed strong positive relationships between phenolic and flavonoid contents and antioxidant indices, underscoring the structure–function link between these bioactives and antioxidant performance. In parallel, antinutritional compounds such as tannins, phytates, oxalates, alkaloids, and saponins were quantified, providing a more complete biochemical profile of pumpkin flowers and enabling species-level comparisons relevant to their nutritional evaluation.

The obtained results highlight the potential of pumpkin flowers—especially *C. maxima* and *C. moschata*—as promising sources of natural antioxidants. These findings offer translational insights for their practical use in functional foods, dietary supplements, and sustainable food processing. Future studies should expand chromatographic profiling to include flavonoids and carotenoids to fully elucidate their contribution to the antioxidant capacity of *Cucurbita* flowers.

## Figures and Tables

**Table 1:** Total Phenolic Content (TPC), Total Flavonoid Content (TFC), Total Phenolic Acid Content (TPAC), Total Carotenoids, and Anthocyanin Content in the Flowers of Five Pumpkin Species.

Species	TPC (mg GAE g^−1^ d.w.)	TFC (mg QE g^−1^ d.w.)	TPAC (mg CAE g^−1^ d.w.)	Total Carotenoids (mg 100 g^−1^ d.w.)	Anthocyanins (mg C3G 100 g^−1^)
Giant pumpkin	47.29 ^a^ ± 0.065	21.32 ^a^ ± 0.04	2.13 ^b^ ± 0.02	38.05 ^d^ ± 0.20	11.73 ^b^ ± 0.05
Summer squash	34.20 ^e^ ± 0.07	17.16 ^d^ ± 0.02	2.11 ^b^ ± 0.05	41.98 ^b^ ± 0.12	9.23 ^d^ ± 0.06
Butternut squash	42.42 ^c^ ± 0.043	19.27 ^c^ ± 0.02	2.17 ^b^ ± 0.05	40.01 ^c^ ± 0.12	12.18 ^a^ ± 0.04
Fig-leaf gourd	35.18 ^d^ ± 0.036	15.70 ^e^ ± 0.04	2.29 ^a^ ± 0.02	48.27 ^a^ ± 0.09	8.48 ^e^ ± 0.07
Cushaw squash	45.62 ^b^ ± 0.03	20.40 ^b^ ± 0.03	2.16 ^b^ ± 0.03	31.35 ^e^ ± 0.07	10.83 ^c^ ± 0.06

Mean values ± standard deviation marked with the same letters in each column do not differ significantly at the significance level α = 0.05. TPC—total phenolic content; TFC—total flavonoids content; TPAC—total phenolic acids content; GAE—gallic acid equivalents, QE—quercetin equivalents, CAE—caffeic acid equivalents, C3G—cyanidin-3-glucoside equivalents; d.w.—dry weight. Data represent mean values ± standard deviation (SD) from three independent replicates (*n* = 3). Mean values followed by the same letter within a row or column do not differ significantly at *p* < 0.05 according to Tukey’s test.

**Table 2:** Characterization of the main phenolic compounds identified in pumpkin flower extracts.

Compound	Retention Time (min)	λmax (nm)	[M–H]^−^ (*m*/*z*)	Fragment Ions (*m*/*z*)	Molecular Formula	Identification Method
Gallic acid	3.2	270	169	125, 97	C_7_H_6_O_5_	HPLC-DAD, LC-MS/MS
Protocatechuic acid	4.6	280	153	109	C_7_H_6_O_4_	HPLC-DAD, LC-MS/MS
p-Hydroxybenzoic acid	6.8	280	137	93	C_7_H_6_O_3_	HPLC-DAD, LC-MS/MS
Caffeic acid	11.2	320	179	135, 107	C_9_H_8_O_4_	HPLC-DAD, LC-MS/MS
Ferulic acid	14.5	322	193	178, 134	C_10_H_10_O_4_	HPLC-DAD, LC-MS/MS
p-Coumaric acid	16.8	310	163	119, 93	C_9_H_8_O_3_	HPLC-DAD, LC-MS/MS
Vanillic acid	18.4	290	167	152, 108	C_8_H_8_O_4_	HPLC-DAD, LC-MS/MS

**Table 3:** Content of Individual Phenolic Acids in Flowers of Five Pumpkin Species.

Phenolic Acids	Giant Pumpkin	Summer Squash	Butternut Squash	Fig-Leaf Gourd	Cushaw Squash
	μg g^−1^ d.w.				
Ferulic acid	19.37 ^a^ ± 0.4	18.72 ^b^ ± 0.6	19.60 ^a^ ± 0.4	17.86 ^c^ ± 0.5	17.27 ^c^ ± 0.4
Gallic acid	19.90 ^b^ ± 0.3	18.41 ^b^ ± 0.6	20.17 ^b^ ± 0.5	23.19 ^a^ ± 0.4	19.47 ^b^ ± 0.7
p-Hydroxybenzoic acid	15.40 ^b,c^ ± 0.6	12.77 ^d^ ± 0.6	15.91 ^b^ ± 0.2	14.43 ^c^ ± 0.5	16.93 ^a^ ± 0.6
Caffeic acid	28.41 ^a^ ± 0.7	26.41 ^a^ ± 0.8	25.61 ^a^ ± 0.6	28.15 ^a^ ± 0.4	27.32 ^a^ ± 0.4
p-Coumaric acid	140.18 ^c^ ± 0.8	146.51 ^b^ ± 0.34	131.57 ^d^ ± 0.30	151.51 ^a^ ± 0.31	124.40 ^e^ ± 0.36
Protocatechuic acid	14.48 ^c^ ± 0.12	15.47 ^b^ ± 0.10	12.22 ^d^ ± 0.10	16.27 ^a^ ± 0.06	15.38 ^b^ ± 0.06
Vanillic acid	8.15 ^c^ ± 0.4	9.28 ^b^ ± 0.5	11.83 ^a^ ± 0.7	12.31 ^a^ ± 0.6	6.80 ^d^ ± 0.2

Mean values ± standard deviation marked with the same letters in each row do not differ significantly at the significance level α = 0.05; d.w.—dry weight Data represent mean values ± standard deviation (SD) from three independent replicates (*n* = 3). Mean values followed by the same letter within a row or column do not differ significantly at *p* < 0.05 according to Tukey’s test.

**Table 4:** Antioxidant properties determined by DPPH, FRAP, ^•^OH CUPRAC tests) of flowers of five pumpkin species.

Species	DPPH (μmol TE g^−1^ d.w.)	FRAP (μmol Fe(II) g^−1^ d.w.)	^•^OH (μmol QE g^−1^ d.w.)	CUPRAC (μmol TE g^−1^ d.w.)
Giant pumpkin	11.50 ^a^ ± 0.4	62.64 ^a^ ± 0.12	97.24 ^a^ ± 0.09	114.52 ± 0.06
Summer squash	10.18 ^c^ ± 0.4	55.30 ^e^ ± 0.08	89.21 ^e^ ± 0.07	110.86 ± 0.08
Butternut squash	10.87 ^b^ ± 0.4	56.20 ^d^ ± 0.08	94.82 ^c^ ± 0.07	111.78 ± 0.10
Fig-leaf gourd	10.67 ^b,c^ ± 0.2	59.83 ^c^ ± 0.05	90.26 ^d^ ± 0.08	112.65 ± 0.07
Cushaw squash	11.22 ^a^ ± 0.6	61.55 ^b^ ± 0.16	96.76 ^b^ ± 0.09	112.88 ± 0.04

Mean values ± standard deviation marked with the same letters in each row do not differ significantly at the significance level α = 0.05; d.w.—dry weight. Data represent mean values ± standard deviation (SD) from three independent replicates (*n* = 3). Mean values followed by the same letter within a row or column do not differ significantly at *p* < 0.05 according to Tukey’s test.

**Table 5:** Pearson’s correlation coefficients between the concentrations of bioactive compounds and the antioxidant capacity.

	DPPH	FRAP	^•^OH	CUPRAC	TPC	TFC	TPAC	Total Carotenoids
FRAP	0.845096							
^•^OH	0.939216	0.651756						
CUPRAC	0.925155	0.934425	0.55101					
TPC	0.940864	0.65946	0.997019	0.72704				
TFC	0.829023	0.515449	0.941635	0.600845	0.959144			
TPAC	−0.08048	0.164558	−0.30258	0.082068	−0.35026	−0.60074		
Total Carotenoids	−0.58202	−0.37229	−0.77522	−0.27521	−0.7748	−0.83785	0.60276	
Anthocyanins	0.670795	−0.37229	0.841529	0.366216	0.841376	−0.48942	−0.48942	−0.62535701

**Table 6:** Content of Antinutritional Compounds in Flowers of Five Pumpkin Species.

Species	Tannins	Phytates	Oxalates	Alkaloids	Saponins
	mg 100 g^−1^ d.w.				
Giant pumpkin	2.10 ^b^ ± 0.02	4.94 ^a^ ± 0.02	0.21 ^b^ ± 0.01	0.26 ^b^ ± 0.02	54.6 ^a^ ± 1.53
Summer squash	1.90 ^d^ ± 0.02	4.50 ^c^ ± 0.01	0.25 ^a^ ± 0.01	0.17 ^d^ ± 0.01	57.7 ^a^ ± 1.53
Butternut squash	2.21 ^a^ ± 0.02	4.17 ^e^ ± 0.03	0.17 ^c^ ± 0.01	0.21 ^c^ ± 0.01	44.7 ^c^ ± 1.53
Fig-leaf gourd	1.98 ^c^ ± 0.02	4.69 ^b^ ± 0.02	0.15 ^d^ ± 0.01	0.31 ^a^ ± 0.02	41.3 ^c^ ± 1.54
Cushaw squash	1.78 ^e^ ± 0.02	4.34 ^d^ ± 0.02	0.20 ^b^ ± 0.01	0.26 ^b^ ± 0.01	49.6 ^b^ ± 0.58

Mean values ± standard deviation marked with the same letters in each row do not differ significantly at the significance level α = 0.05; d.w.—dry weight. Data represent mean values ± standard deviation (SD) from three independent replicates (*n* = 3). Mean values followed by the same letter within a row or column do not differ significantly at *p* < 0.05 according to Tukey’s test.

## Data Availability

The original contributions presented in this study are included in the article. Further inquiries can be directed to the corresponding author.
